# Pressure cycling technology-assisteddata-independent acquisition proteomics reveals molecular alterations and potential therapeutic targets in minor glomerular abnormalities

**DOI:** 10.1093/pcmedi/pbag006

**Published:** 2026-02-13

**Authors:** Ling Li, Yingying Ling, Fei Cai, Yi Zhong, Hao Yang, Fang Liu, Guisen Li, Xinfang Xie, Rajeev K Singla, Dengyan Ma, Yong Zhang

**Affiliations:** Department of Nephrology, Kidney Research Institute, West China Hospital, Sichuan University, Chengdu 610041, China; Department of Nephrology, Institutes for Systems Genetics, Frontiers Science Center for Disease-related Molecular Network, West China Hospital, Sichuan University, Chengdu 610041, China; Department of Nephrology, Institutes for Systems Genetics, Frontiers Science Center for Disease-related Molecular Network, West China Hospital, Sichuan University, Chengdu 610041, China; Department of Nephrology, Institutes for Systems Genetics, Frontiers Science Center for Disease-related Molecular Network, West China Hospital, Sichuan University, Chengdu 610041, China; Department of Nephrology, Institutes for Systems Genetics, Frontiers Science Center for Disease-related Molecular Network, West China Hospital, Sichuan University, Chengdu 610041, China; Department of Nephrology, Kidney Research Institute, West China Hospital, Sichuan University, Chengdu 610041, China; Renal Department and Institute of Nephrology, Sichuan Provincial People’s Hospital, University of Electronic Science and Technology of China, Sichuan Clinical Research Center for Kidney Diseases, Chengdu 611731, China; Department of Nephrology, The First Affiliated Hospital of Xi’an Jiaotong University, Xi’an 710061, China; Department of Pharmacy and Institutes for Systems Genetics, Center for High Altitude Medicine, Frontiers Science Center for Disease-related Molecular Network, West China Hospital, Sichuan University, Chengdu 610041, China; School of Pharmaceutical Sciences, Lovely Professional University, Phagwara, Punjab 144411, India; Department of Nephrology, Kidney Research Institute, West China Hospital, Sichuan University, Chengdu 610041, China; Department of Nephrology, Kidney Research Institute, West China School of Nursing, Sichuan University, Chengdu 610041, China; Department of Nephrology, Kidney Research Institute, West China Hospital, Sichuan University, Chengdu 610041, China; Department of Nephrology, Institutes for Systems Genetics, Frontiers Science Center for Disease-related Molecular Network, West China Hospital, Sichuan University, Chengdu 610041, China

**Keywords:** kidney tissue, minor glomerular abnormality, distant non-neoplastic tissue, proteomics, pressure cycling technology, LC-MS/MS

## Abstract

**Background:**

Minor glomerular abnormalities (MGAs) are histopathologically heterogeneous renal lesions with subtle structural changes and latent clinical manifestations, yet their molecular mechanisms remain poorly characterized and underexplored.

**Methods:**

In this study, we employed pressure cycling technology-assisted sample preparation combined with data-independent acquisition mass spectrometry to systematically compare the proteomic profiles of distant non-neoplastic tissues (*n* = 24) and MGA tissues (*n* = 27).

**Results:**

A total of 9 529 protein groups were quantified with a false discovery rate < 1%, and 1 338 differentially expressed protein groups were identified (fold-change > 2 or < 0.5, *P* < 0.05), including 190 downregulated and 1 148 upregulated protein groups in MGA tissues. Gene ontology analysis revealed that the downregulated proteins were enriched in cell adhesion, ion binding, and molecular transport, whereas the upregulated proteins were enriched in transcriptional regulation, DNA replication/repair, and nucleic acid binding. Kyoto Encyclopedia of Genes and Genomes pathway analysis indicated inhibition of metabolic pathways and the peroxisome proliferator-activated receptor signaling pathway, as well as the activation of basal transcription factors and nucleotide excision repair in MGAs. Further screening revealed 13 core upregulated nuclear proteins (e.g. YY1, TAF9, RFC1, and POLR1D) with a >90% detection rate in MGA tissues; these proteins are functionally associated with renal inflammation, cell proliferation, and the DNA damage response.

**Conclusion:**

Our study establishes a high-resolution proteomic landscape of MGAs, provides novel insights into their molecular pathogenesis, and identifies potential tissue biomarkers and therapeutic targets. The pressure cycling technology-assisted data-independent acquisition workflow also offers a robust technical framework for proteomic analysis of microscale renal biopsy samples.

## Graphical Abstract

**Graphical Abstract:**
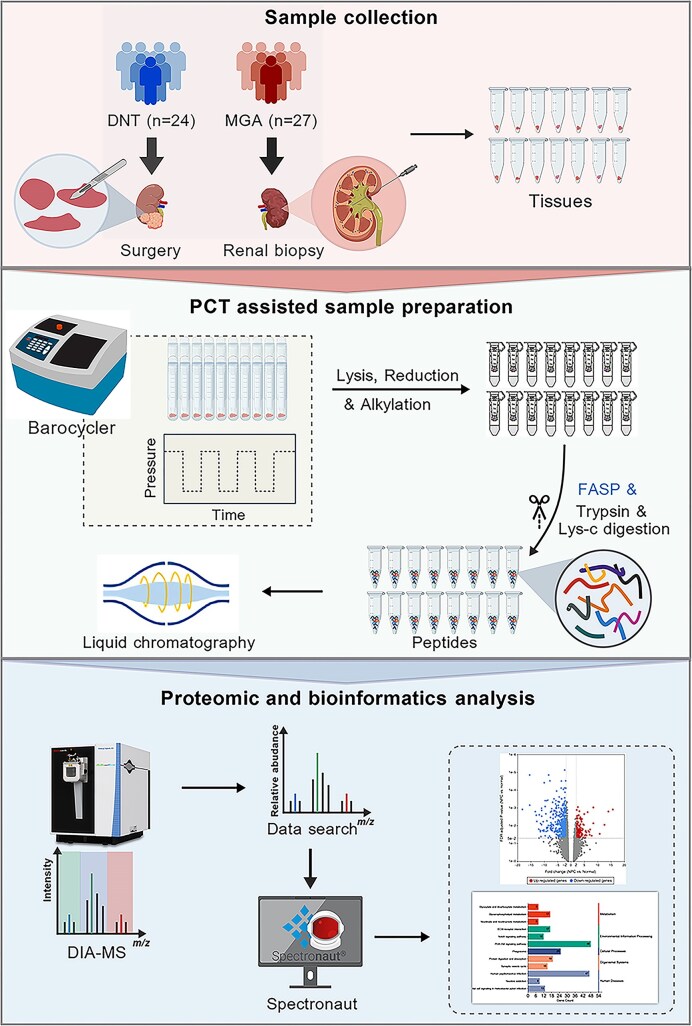
Schematic representation of the analysis workflow for PCT-assisted DIA proteomics.

## Introduction

Minor glomerular abnormalities (MGAs), alternatively termed mild glomerular lesions, refer to a group of glomerular diseases characterized by mild pathological changes and clinical manifestations of latent nephritis, and represent a histopathological entity characterized by subtle structural alterations in glomeruli that defy conventional classification [1]. Although identifiable by light microscopy, electron microscopy, or immunofluorescence, these lesions often lack diagnostic specificity despite their clinical significance [2]. MGAs encompass a spectrum of mild glomerular disorders, including minimal change disease (MCD), mild mesangial proliferative glomerulonephritis, stage Ⅰ membranous nephropathy, and a variety of secondary glomerular diseases with subtle pathological changes. Clinically, MGAs typically present as latent nephritis and account for ∼3.8% of all renal biopsies in adults. This detection rate (DR) increases markedly in patients with isolated urinary abnormalities, reaching 5%–8% in those with persistent isolated proteinuria and 14.5%–25% in those with isolated microscopic hematuria [1–3].

Emerging evidence suggests a complex clinical trajectory for patients with MGAs. While some patients exhibit indolent progression, longitudinal studies have documented associations with declining renal function and ultrastructural mitochondrial damage [4, 5]. These observations underscore the imperative for systematic patient monitoring. Paradoxically, despite their diagnostic frequency, MGAs remain molecularly enigmatic: protein-level aberrations are poorly characterized, and evidence-based management protocols are conspicuously absent. To address these gaps, we employed high-resolution mass spectrometry (MS) to delineate renal proteomic signatures in MGAs.

Contemporary MS-based proteomics has revolutionized disease mechanism elucidation, biomarker discovery, and therapeutic target identification [6–8]. However, renal tissue proteomic studies face unique ethical and methodological challenges, particularly with respect to controlling tissue procurement. While distant non-neoplastic tissues (DNTs) from tumor-adjacent regions remain the conventional control [9, 10], some investigators controversially utilize MGA tissues [11–13]. This methodological discordance highlights the urgent need for standardized proteomic reference frameworks. Furthermore, given the minimal tissue volume typically obtained from kidney biopsies, it is essential to employ highly efficient protein extraction protocols and low-waste sample preparation methods to yield adequate peptides for MS analysis [14].

Our study pioneers a systematic comparison of the DNT and MGA proteome using cutting-edge analytical workflows. Freshly obtained kidney tissues (acquired via puncture or surgery) were subjected to pressure cycling technology (PCT)-assisted lysis [14, 15]. The extracted proteins then underwent proteolysis via a modified filter-aided sample preparation (FASP) and data-independent acquisition (DIA) MS. This approach not only establishes rigorous methodological benchmarks for control selection but also maps MGA-specific protein networks, potentially revealing actionable biomarkers or therapeutic targets. By integrating advanced proteomic technologies with clinical nephrology, this work may redefine diagnostic paradigms and therapeutic strategies for MGAs.

## Methods

### Materials and chemicals

Tris(2-carbonylethyl)phosphine, iodoacetamide (IAA), trifluoroacetic acid, formic acid (FA), urea, thiourea, and ammonium bicarbonate (NH_4_CO_3_) were purchased from Sigma (St. Louis, MO, USA). Acetonitrile (ACN) was obtained from Merck (Darmstadt, Germany). The electrophoresis reagents were supplied by Bio-Rad (Richmond, CA, USA). Sequencing-grade trypsin and Lys-C were purchased from Enzyme & Spectrum (Beijing, China). The Bradford protein assay kit, quantitative colorimetric peptides assay kit, and molecular weight markers were obtained from Thermo Fisher Scientific (Rockford, IL, USA). The 30-kDa centrifugal filters were obtained from Merck Millipore (Carrigtwohill, Ireland). All other chemicals and reagents were purchased from Sigma-Aldrich (St. Louis, MO, USA).

### Biospecimen collection

Human kidney tissue samples were collected from two groups. The first group consisted of DNTs (*n* = 24), which were obtained from patients who underwent unilateral nephrectomy at the Department of Urology, West China Hospital of Sichuan University, China. The second group included MGA (*n* = 27) tissues collected from patients who underwent renal biopsy at the Department of Nephrology, West China Hospital of Sichuan University, China. All the samples were stored in 1.5 ml tubes at −80°C. The study complied with the principles of the Declaration of Helsinki and was approved by the Ethics Committee of West China Hospital of Sichuan University, China [Approval No. 2024(1221)]. All participants provided written informed consent. There were two categories of participants in this study, with the grouping and inclusion criteria detailed as follows. The DNT group included patients who required unilateral radical nephrectomy due to renal space-occupying lesions. The inclusion criteria were: (i) no history of blood-borne infectious diseases, such as hepatitis B, syphilis, or human immunodeficiency virus infection; and (ii) tissue samples must be collected from locations at least 5 cm away from the lesion edge, with pathological examination confirming that no tumor-cell infiltration has occurred.​

Patients were included in the MGA group on the basis of the simultaneous fulfillment of the following criteria: (i) the pathological diagnosis aligned with internationally standardized diagnostic criteria for mild glomerular lesions; (ii) immunological tests returned negative results for serum complement components (C3 and C4), antinuclear antibody, anti-double stranded DNA antibody, and the extractable nuclear antigen antibody profile; (iii) detection results of serum protein electrophoresis, serum immunofixation, and the serum free light chain κ/λ ratio were all negative; (iv) no history of infectious diseases such as hepatitis B, hepatitis C, syphilis, or human immunodeficiency virus infection; and (v) no evidence of complicated malignant tumors was found before surgery or during the follow-up period. For all patients, renal biopsy specimens were required to contain >10 glomeruli. Following tissue processing, light microscopy, immunofluorescence, and electron microscopy examinations were performed. The pathological criteria were as follows. (i) Light microscopy findings: the glomeruli mostly exhibit normal morphology, with no significant alterations, but may display mild mesangial cell proliferation or a slight increase in the mesangial matrix. (ii) Immunofluorescence findings revealed an absence of immunoglobulin deposition. (iii) Electron microscopy findings: the glomeruli basically exhibit a normal morphology, or they may be accompanied by extensive or partial fusion of foot processes or thinning of the basement membrane. There is no electron-dense deposit in the glomeruli, or only a small amount of electron-dense deposit present in the individual mesangial areas of the glomeruli. Diagnostic confirmation for all samples was established through standard pathological assessment, including hematoxylin and eosin (H&E) staining, by an experienced renal pathologist.

### Histopathological examination and H&E staining

For conventional histopathological evaluation, portions of the collected kidney tissue samples were fixed in 10% neutral-buffered formalin for 24–48 h, followed by standard dehydration, paraffin embedding, and sectioning at 4 μm thickness. H&E staining was performed according to established protocols. Briefly, tissue sections were deparaffinized in xylene and rehydrated through a graded ethanol series to distilled water. Nuclei were stained with Harris hematoxylin for 5–8 min, followed by differentiation in 1% acid alcohol and bluing in 0.2% ammonia water. Cytoplasmic and extracellular matrix components were then counterstained with eosin Y solution for 1–3 min. After dehydration through graded ethanol and clearing in xylene, the sections were mounted with neutral balsam. All stained slides were reviewed and diagnosed by an experienced renal pathologist to confirm the histopathological features of MGA (e.g. preserved glomerular architecture without significant hypercellularity or sclerosis) and to verify the absence of tumor cells in DNTs. Representative digital images were captured using a light microscope (Olympus BX53) equipped with a high-resolution camera at 50 × magnification.

### PCT-assisted tissue lysis

The kidney tissue samples obtained were subjected to a PCT-assisted tissue lysis procedure. Each tissue sample (<1 mg) was placed into a PCT-MicroTube and suspended in 50 μl of lysis buffer (6 M urea, 2 M thiourea, 100 mM NH_4_CO_3_, 20 mM TCEP, and 40 mM IAA). The samples were then processed via a Barocycler (Pressure Biosiciences, USA) with a cycle setting of 90, which involved exposure to 45 000 psi for 30 s and ambient pressure for 10 s, at a temperature of 30°C. Next, the lysate was transferred to a 1.5 ml tube, where 100 μl of 8 M urea was added as a supplement. The protein concentration was determined by using a protein assay kit.

### Protein reduction, alkylation, and digestion

The extracted proteins were subjected to proteolysis via a modified FASP method. For this process, proteins were placed in a 30-kDa filter and subjected to centrifugation at 25 °C for 15 min at 13 000 g. The UA buffer (8 M urea) was replaced with 50 mM NH_4_CO_3_ buffer through centrifugation at 13 000 g for three cycles of 15 min each. Trypsin and Lys-C (1 μg of each) were subsequently added to the mixture for digestion overnight at 37°C. To obtain the peptides, the solution was again centrifuged at 13 000 g for 15 min. The concentration of these peptides was determined via a quantitative colorimetric peptide assay at 480 nm, followed by freeze-drying with a SpeedVac.

### Independent validation by Western blotting

To biochemically validate the proteomic findings, we performed western blot analysis on an independent cohort of kidney tissues. This validation cohort consisted of 3 randomly selected DNT samples and 3 MGA samples that were not used in the initial PCT-DIA proteomics discovery phase. Tissue lysates were prepared utilizing RIPA lysis buffer containing 1% protease and phosphatase inhibitors. Total protein was extracted, protein concentration was quantified using the BCA protein quantification assay kit, and the protein concentration of each sample was adjusted to 1 µg per µl with 5 × SDS loading buffer. Then, they were heated at 95 °C for 10 min. The proteins were loaded on a 10% separation gel, and the gel was run in SDS electrophoresis buffer at 80 V for 30 min and 120 V for 1 h. Equal amounts of protein (10 μg per lane) were separated by 10% SDS-PAGE and transferred to PVDF membranes. These membranes were blocked with 5% nonfat milk at room temperature for 1 h before being incubated overnight at 4°C with the following primary antibodies: anti-Yin Yang 1 (YY1) rabbit monoclonal antibody (1:1000, ZENBIO, R26129), anti-TATA-box binding protein associated factor 9 (TAF9) rabbit monoclonal antibody (1:1000, Abclonal, A25852), and anti-switch/sucrose non-fermentable chromatin remodeling complex (SWI/SNF)-related, matrix associated, actin dependent regulator of chromatin, subfamily D, member 1 (SMARCD1) rabbit monoclonal antibody (1:1000, ZENBIO, R27292). GAPDH (1:100 000, Abclonal, A19056) was used as a loading control. The membranes were then washed three times with TBST (10 min each) before being incubated with HRP-conjugated secondary antibodies (1:10 000) for 60 min. Following this, the membranes were washed three times with TBST and visualized utilizing chemiluminescence with an ECL detection system. The relative protein expression levels were then quantified using Image J software. Statistical significance was assessed using an unpaired two-tailed Student’s t-test.

### Liquid chromatography tandem MS analysis

Peptide analysis was conducted via an Orbitrap Exploris 480 mass spectrometer (Thermo Fisher, USA) connected to a Vanquish Neo system with a nanospray ion source. The liquid chromatography (LC) gradient used buffer A (0.1% FA) and buffer B (80% ACN with 0.1% FA). Peptides were dissolved in 20 μl of buffer A, and a 2 μl sample was injected for separation on a self-packed analytical column measuring 25 cm in length and 75 µm inner diameter. This column was filled with ReproSil-Pur 120 C18-AQ resin (1.9 µm, 120 Å, Dr. Maisch, Germany) and maintained at 55°C. Peptide separation occurred over a 78-min gradient at a flow rate of 300 nl/min, with the following buffer B conditions: 3%–5% from 0 to 1 min; 5%–20% from 1 to 49 min; 20%–35% from 49 to 73 min; 35%–95% from 73 to 73.5 min; and 95% from 73.5 to 78 min. The peptides were analyzed via a DIA technique. MS1 spectra were collected within a 350–1400 *m*/*z* range at a resolution of 120 000. The RF lens, AGC target, and maximum ion time (MIT) were set to 50%, custom, and 25 ms, respectively. MS2 spectra were captured with a 1.6 *m*/*z* isolation width at a resolution of 30 000. The AGC target, MIT, activation type, and collision energy settings were custom, 40 ms, higher-energy collisional dissociation, and 32%, respectively. For quality control (QC) of the performance of MS, the HeLa cell lines lysate, as the QC standard, was measured every 10 samples.

### Data analysis and bioinformatics

Spectronaut software (v15.4, Biognosys) was used to analyze the DIA data files against the human UniProt database (version 2015_03, 20 410 entries). Unless specified otherwise, the software was operated using its default settings. Filtering was executed based on the “Qvalue” criterion. The mass tolerance for precursor and fragment ions was maintained at ±10 ppm and ±20 ppm, respectively, allowing for up to two missed cleavage sites. Carbamidomethylation of cysteine residues was designated as a fixed modification, whereas variable modifications included methionine oxidation and N-terminal acetylation of proteins. We filtered protein groups to ensure a 1% false discovery rate (FDR). Data analysis was performed via R programming software in conjunction with our proprietary Wukong platform. Specifically, PGs with a missing value rate >50% were excluded. Remaining missing values were imputed using the minimum value across the entire proteomic dataset. Next, the median intensity of each sample was calculated, and the intensity of each protein in one sample was normalized to this median to eliminate systematic variations between samples. Finally, logarithmic transformation was performed prior to statistical analysis. Differences were considered statistically significant for *P* < 0.05. A fold-change (FC) > 2 was defined as upregulated expression, while an FC < 0.5 indicated downregulated expression. Pearson correlation analysis was used to assess the correlation between samples; strong positive or negative correlations were defined as *r* > 0.6 or < −0.6, respectively.

## Results

### Histopathological characteristics of study cohorts

To confirm the pathological diagnosis and tissue integrity of our study samples, representative renal tissue sections from both the MGA and DNT groups were subjected to H&E staining. As shown in Supplementary Fig. 1 (see online supplementary material), H&E staining of MGA tissues revealed largely preserved glomerular architecture. The glomeruli exhibited no significant mesangial hypercellularity, capillary wall thickening, or glomerulosclerosis, which is consistent with the defining histopathological features of MGAs. Conversely, H&E staining of DNT samples, obtained from regions distant to renal tumors, demonstrated intact renal parenchyma with preserved glomerular and tubular architecture, and crucially, no evidence of malignant cell infiltration. These findings confirm the appropriateness of our sample classification and provide the morphological context for the subsequent molecular profiling.

### PCT-assisted quantitative proteomic workflow in human kidney tissues

**Figure 1:**
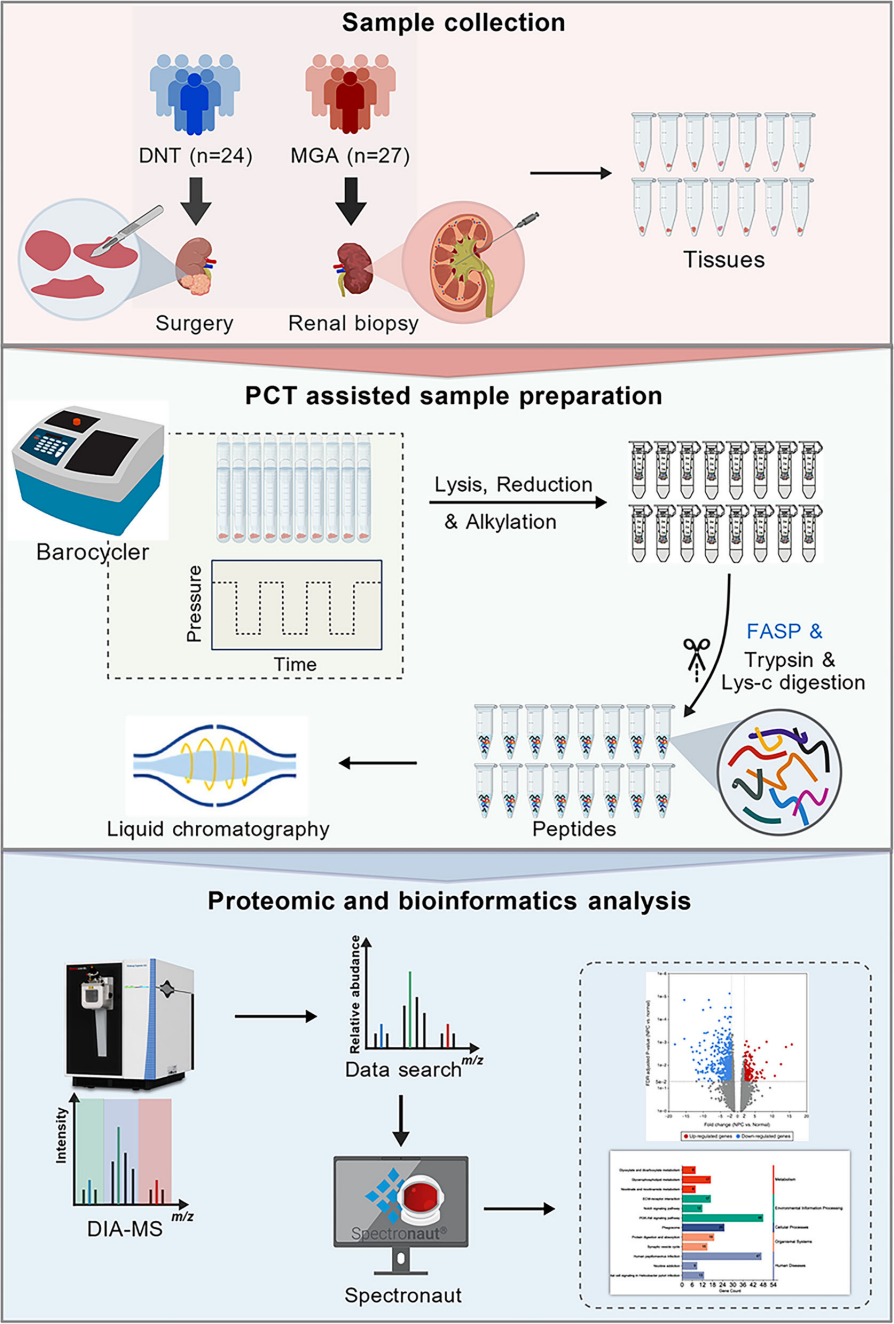
Schematic representation of the analysis workflow for PCT-assisted DIA proteomics. This figure was created with BioGDP (https://BioGDP.com).

Renal biopsy tissue analysis remains the gold standard for diagnosing most kidney diseases. While a portion of collected kidney tissue is reserved for standard pathological examination, the residual material available for research is often severely limited [16]. Conventional protein extraction methods, including pestle grinding, glass homogenization, and bead milling, frequently result in suboptimal protein yields and substantial protein losses [17, 18]. To address these limitations, we implemented PCT for proteomic preparation of renal biopsy specimens (Fig. 1). PCT represents an innovative sample-processing approach utilizing a barocycler device to apply ultrahigh hydrostatic pressure (up to 45 000 psi) [15]. Through rapid cycling between elevated and ambient pressures, this method achieves efficient physical disruption of complex kidney tissues, enabling superior protein extraction and solubilization [19]. Notably, obtaining adequate protein is beneficial for subsequent research on post-translational modifications [14]. In this study, kidney tissue samples were collected from two groups. There were 24 DNTs from patients who underwent unilateral nephrectomy in the Department of Urology. Given the ethical challenges of procuring healthy human kidney tissues, DNTs are widely recognized as suitable controls in kidney disease-based research [20–22]. Another 27 tissues with MGAs were obtained via renal biopsy in the Department of Nephrology. Supplementary Table 1 (see online supplementary material) provides a detailed summary of the baseline characteristics of the DNT and MGA groups. Following protein extraction via PCT, we integrated FASP with DIA-MS (Fig. 1). This pipeline enables comprehensive protein profiling, offering protein-level insights into MGA mechanisms and revealing potential therapeutic targets. Modern MS-based quantitative proteomics has revolutionized nephrological research by allowing high-throughput identification and quantification of thousands of proteins across tissue samples [23]. This advanced methodology could provide invaluable insights for both clinical diagnostics and therapeutic development for kidney diseases.

### Quantitative proteomic profiles in human kidney tissues

Prior to quantitative analysis, we performed extensive QC to validate the robustness of our proteomic dataset. The HeLa cell lysate was used as a QC standard and analyzed every 10 experimental samples (supplementary Table 2, see online supplmentary material). Base peak intensity chromatograms derived from the six QC sample datasets exhibited consistent retention times and signal intensities across all replicates (supplementary Fig. 2A, see online supplementary material). The Pearson correlation heatmap of quantified protein expression levels across the six replicates confirmed high analytical reproducibility with all correlation coefficients >0.95 (supplementary Fig. 2B). Additionally, a Pearson correlation heatmap of protein expression levels across biological replicates confirmed high reproducibility (supplementary Fig. 3, see online supplementary material). These results affirm the high quality and reliability of the data for subsequent differential expression analysis.

Using the PCT-assisted DIA proteomics strategy, we quantified a total of 9 529 protein groups (PGs) (FDR < 1%). To ensure reliability, PGs with a DR < 50% across samples were excluded, resulting in 7 669 quantified PGs and 1 338 differentially expressed protein groups (DEPGs) (FC > 2 or < 0.5, *P* < 0.05) (Fig. 3A and supplementary Table 3, see online supplementary material). Our quantitative analysis also revealed noteworthy differences in data variability between the sample groups. Specifically, the number of PGs detected exhibited greater inter-sample variability within the DNT group compared to the MGA group (Fig. 3B). This observation raises important methodological considerations: while histopathological examination confirmed normal kidney tissue morphology in the DNT group, the pronounced heterogeneity in protein expression levels suggests that using DNT as a control group may introduce unintended variability. These findings indicate that DNT controls could compromise experimental reliability due to underlying biological fluctuations not reflected in structural assessments.

### Differentially expressed proteins in MGA patients

We performed a comprehensive analysis of DEPGs between the two groups. The volcano plot revealed a striking disparity in protein expression profiles, with 190 significantly downregulated and 1 148 upregulated PGs identified in the MGA group (Fig. 4A and supplementary Table 4, see online supplementary material). Principal component analysis (PCA) of these DEPGs demonstrated clear separation between the two groups (Fig. 4B), indicating distinct protein expression patterns. These findings provide compelling evidence that the two groups exhibit fundamental differences in protein abundance levels. Gene ontology (GO) analysis revealed distinct functional patterns between downregulated and upregulated proteins. The downregulated proteins were mainly localized to extracellular exosome, cytoplasm, and membrane, participating in biological processes including cell adhesion, molecular transport, and cellular response. Functionally, they were enriched for activities such as ion binding and protein-related functions and performed a variety of molecular functions, such as ion binding and protein activity (Fig. 4C). In contrast, the upregulated proteins primarily localized to the nucleus and cytoplasm, and were involved in transcriptional regulation, cellular signaling pathways, and other regulatory processes. Their molecular functions prominently featured nucleic acid (DNA/RNA) binding and metal-ion interactions (Fig. 4D).

Kyoto Encyclopedia of Genes and Genomes (KEGG) pathway analysis further demonstrated significant pathway modulation: 9 pathways showed inhibition, most notably metabolic pathways, along with pathways such as protein digestion and absorption, and the peroxisome proliferator-activated receptor (PPAR) signaling pathway (Fig. 4A), while 5 pathways exhibited activation (Fig. 4B). The activated pathways included basal transcription factors, ATP-dependent chromatin remodeling, RNA polymerase activity, ribosome biogenesis in eukaryotes, and nucleotide excision repair (Fig. 4C). Protein–protein interaction (PPI) network analysis confirmed robust interaction networks among these upregulated proteins, indicating high-confidence functional relationships (Fig. 4C).

### Upregulated key proteins in MGA patients

Furthermore, we analyzed the upregulated proteins exhibiting >90% DRs in MGA patients. This analysis identified 13 significantly upregulated proteins [methyl-CpG binding domain protein 3 (MBD3), RNA polymerase I subunit D (POLR1D), YY1, reduced folate carrier 1 (RFC1), general transcription factor IIA subunit 2 (GTF2A2), ribonuclease P protein subunit p30 (RPP30), TAF9, B-cell CLL/lymphoma 7C (BCL7C), SMARCD1, actin-related protein 6 (ACTR6), DNA methyltransferase associated protein 1, WD repeat domain 3 (WDR3), and adenylate kinase 6 (AK6)]. Notably, all retained proteins were nucleoproteins characterized by low tissue specificity. These proteins undergo ubiquitination and phosphorylation modifications and are functionally associated with nucleic acid binding. Their differential expression patterns across the DNT and MGA groups are illustrated in Fig. 5. Previous studies have indicated that the abnormal expression or modification of nuclear proteins may be involved in the pathogenesis of renal diseases and may affect disease progression [24, 25]. In this study, the functions of these upregulated nuclear proteins were enriched in core biological processes, including transcriptional regulation, DNA replication and repair, and nucleotide metabolism.

### Independent biochemical validation of key upregulated proteins

To provide orthogonal confirmation of our proteomic discoveries, we performed western blot analysis on three selected core proteins (YY1, TAF9, and SMARCD1) using an independent set of kidney tissue samples (*n* = 3 per group). The results consistently demonstrated significantly higher protein levels of YY1 and TAF9 in MGA tissues compared to DNT controls (*P* < 0.05) (Fig. 6A). Densitometric quantification normalized to GAPDH confirmed these observations, revealing increases that were concordant with the MS-based FCs (Fig. 6B). For instance, YY1 and TAF9 showed particularly strong validation, with western blot quantification mirroring their pronounced upregulation in the proteomic data. This independent biochemical validation strongly supports the reliability of our PCT-DIA quantification and underscores the relevance of these nuclear proteins in the pathophysiology of MGAs.

## Discussion

**Figure 2:**
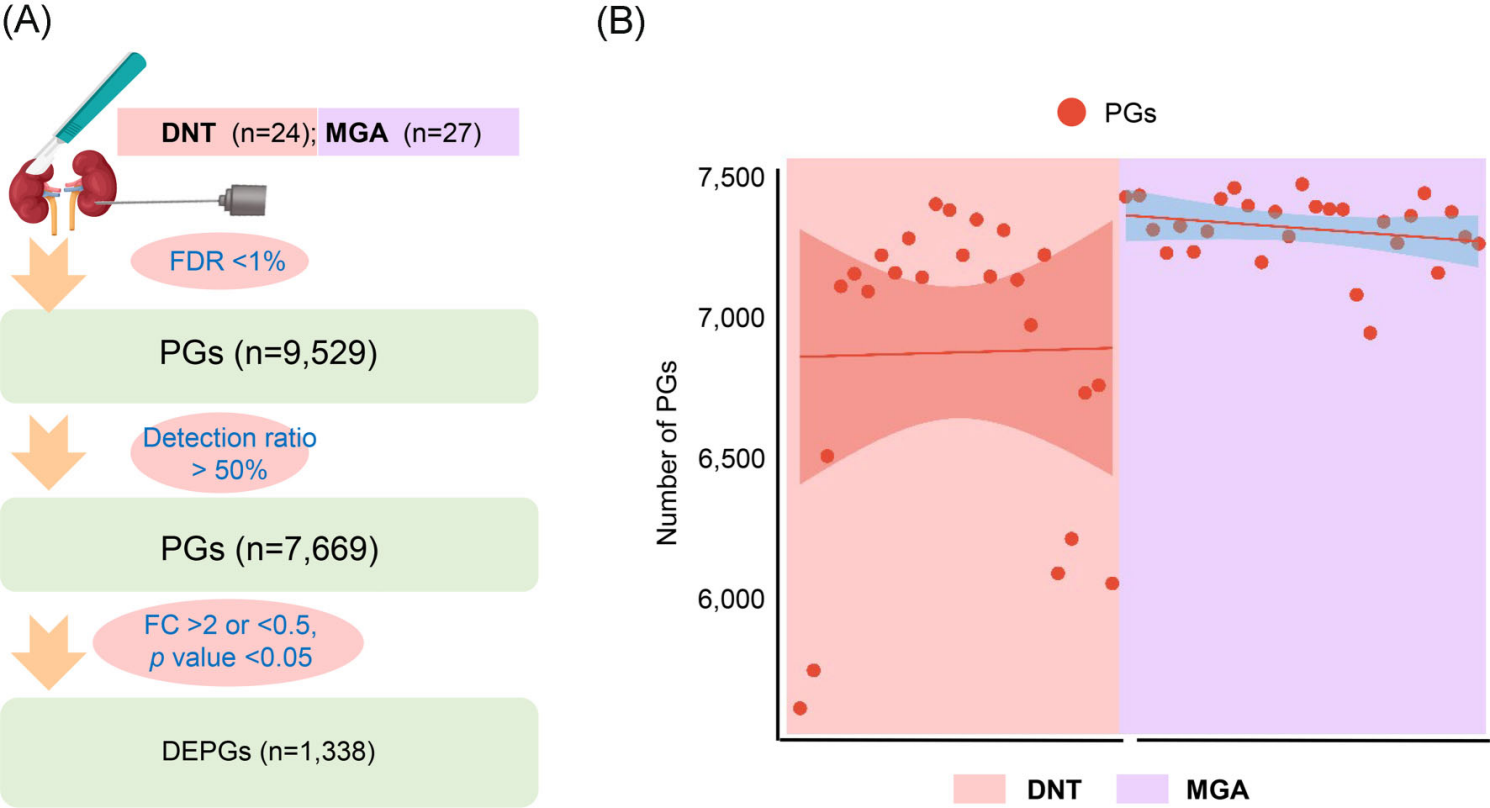
Quantitative proteomic profiles in human kidney tissues. (**A**) Numbers of protein groups quantified in the two groups. (**B**) Quantified amounts of protein groups in each sample.

**Figure 3:**
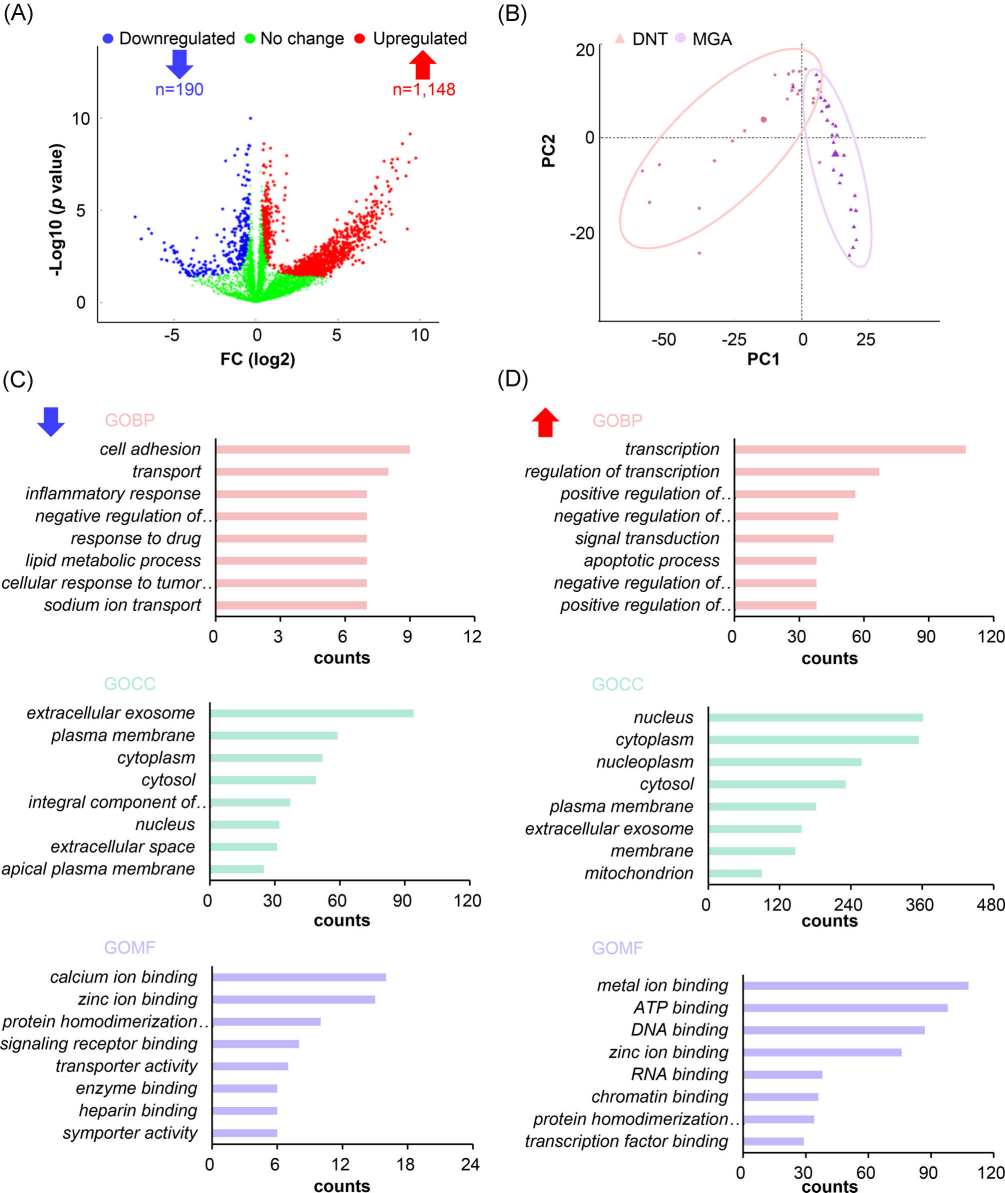
Comparison of quantified proteins between the DNT and MGA groups. (**A**) Volcano plot of DEPGs. (**B**) Principal component analysis plot of the first two components of DEGPs. (**C**) Gene ontology analysis of downregulated DEPGs. (**D**) Gene ontology analysis of upregulated DEPGs. Abbreviations: GOBP, gene ontology biological process; GOCC, gene ontology cellular component; GOMF, gene ontology molecular function; PC, principal component.

**Figure 4:**
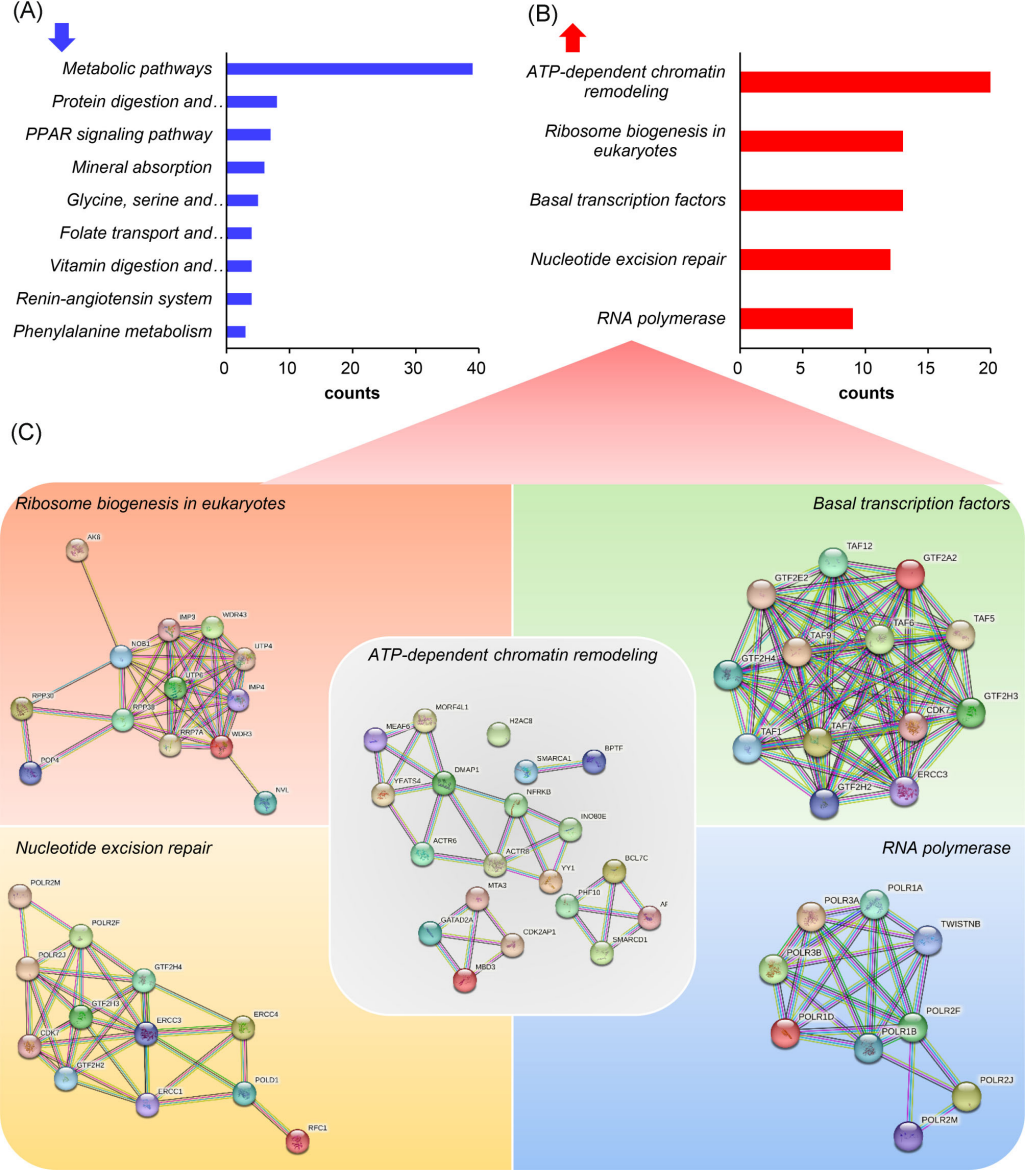
KEGG pathway enrichment analysis. (**A**) Pathways enriched by downregulated PGs. (**B**) Pathways enriched by upregulated PGs. (**C**) PPI analysis of upregulated PGs.

**Figure 5:**
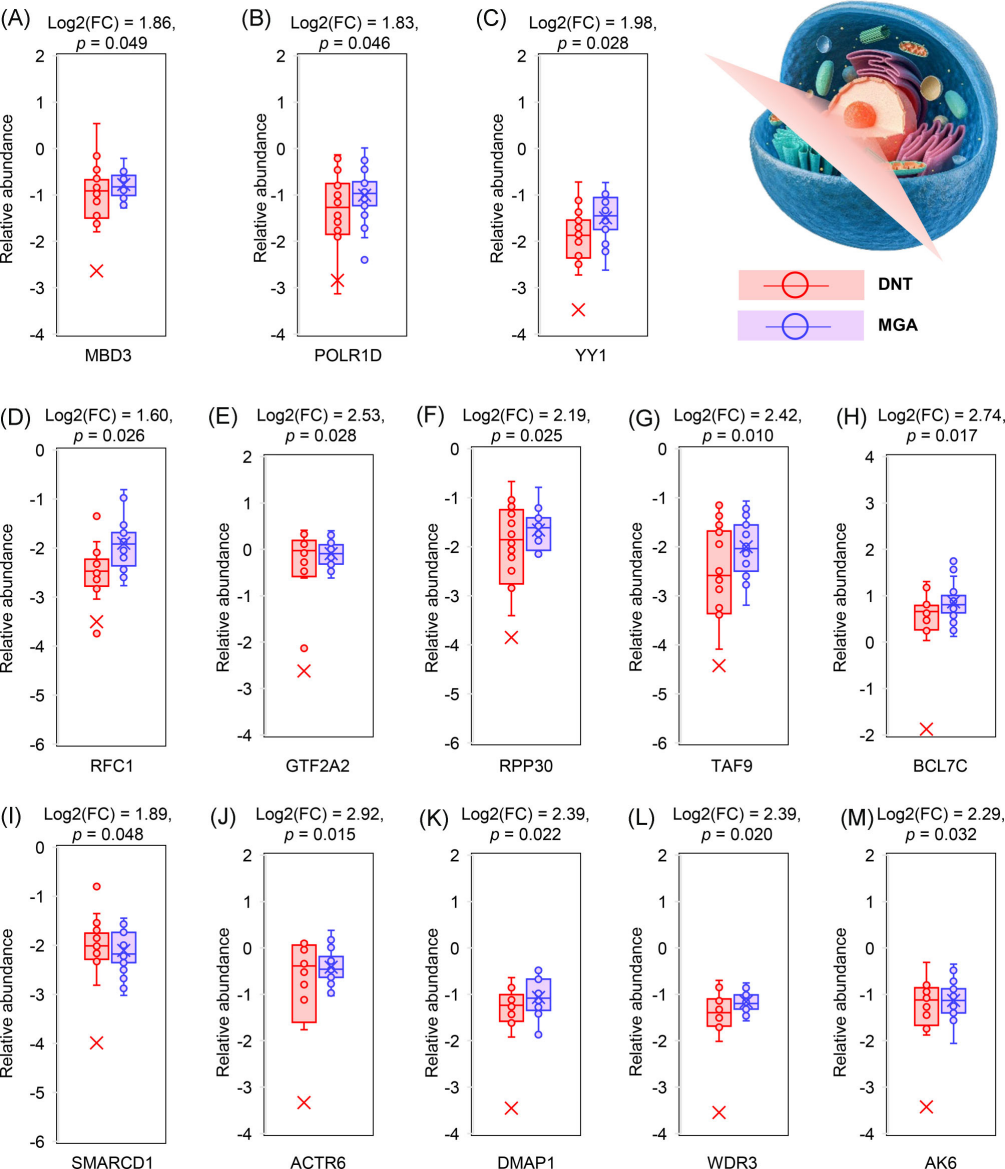
(A–M) Upregulated key proteins in MGA tissues.

Our study established a comprehensive proteomic landscape of MGAs via a cutting-edge workflow integrating PCT-assisted sample preparation and DIA MS (Fig. 1). This approach addressed key technical bottlenecks in microscale renal tissue proteomics, enabling us to quantify nearly 10 000 PGs and identify >1 300 DEPGs in MGAs (Fig. 2). Functional and pathway analyses revealed core molecular features of MGA pathogenesis: downregulated DEPGs are enriched in functions critical for glomerular filtration barrier integrity (cell adhesion, ion binding, and molecular transport), while upregulated DEPGs cluster in transcriptional regulation, DNA replication/repair, and nucleotide metabolism (Figs. 3 and 4), with 13 core nuclear proteins (e.g. YY1, TAF9, RFC1, and POLR1D) showing high DRs in MGAs and linking to renal inflammation, injury adaptation, and pathological proliferation (Figs. 4 and 5). Clinically, these 13 nuclear proteins fill the gap in MGA tissue biomarkers and provide promising therapeutic targets. The reliability of these core findings relies on the technical innovation of the PCT-assisted DIA workflow employed in this study.

A critical advantage of the PCT-assisted DIA workflow underpinning these findings is its superior protein coverage and extraction efficiency compared to conventional methods for renal tissue proteomics. For example, while formalin-fixed paraffin-embedded renal tissue is the most common clinical sample, a modified kit combined with ultrasonic extraction enables the identification of only 840 proteins [26]. Even in acute kidney injury (AKI) after liver transplantation, with the FASP technique, which is optimized specifically for biopsy samples, the number of quantified proteins is only 6 620 [27]. The limitations of non-PCT methods are further exacerbated when microscale or purified tissue regions are analyzed. For example, laser capture microdissection combined with conventional lysis identified only 5 834 proteins in glomeruli, and when laser capture microdissection was coupled with solid phase protein preparation magnetic bead enrichment, only 321 proteins were detected in MCD samples [9, 21]. Collectively, these comparisons demonstrate that non-PCT methods struggle to reconcile protein coverage with the utilization efficiency of previous microscale samples, a core challenge that our PCT-assisted strategy effectively overcomes. The superior performance of our method is also reflected in the depth of differential expression analysis. The number of DEPGs identified in our study was significantly greater than that obtained by non-PCT methods [28]. This provides a more abundant pool of candidate molecules for the screening of kidney disease biomarkers and the exploration of therapeutic targets, ensuring the potential translational value of our results. In summary, the PCT-assisted DIA technology employed herein effectively addresses key bottlenecks in the proteomic analysis of microscale renal tissues, establishing a powerful platform for mechanistic discovery and clinical translation.

This enhanced technical performance also translated to deeper insights into the molecular features of MGA, starting with the functional patterns of DEPGs. The downregulation of cell-adhesion-related proteins aligns with pathological observations in MGAs, such as podocyte foot process fusion: adhesion plaque components mediate the anchoring of podocytes to the glomerular basement membrane, and their reduced expression impairs this attachment, exacerbating structural damage to the filtration barrier [21]. Furthermore, the enrichment of calcium-ion binding and protein-binding functions in our data resonates with findings from other glomerulopathies: in membranous nephropathy (MN), calcium-ion binding cooperates with complement activation and cell adhesion to exacerbate glomerular filtration barrier damage [29]; IgA nephropathy is jointly enriched with serine-type endopeptidase inhibitor activity and complement activation. Thus, the dysfunction of ion-binding-related functions emerges from our analysis as a common driver of filtration barrier damage across glomerular diseases [30]. In contrast, the upregulated proteins focused on transcriptional regulation and DNA repair, reflecting a molecular adaptive response to renal injury. This pattern mirrors findings in MCD (a core MGA subtype), where transforming growth factor beta 1-regulated downstream proteins rely on nucleic acid binding for gene expression [21]. In focal segmental glomerulosclerosis, DEPGs are also involved in the positive regulation of DNA binding [31].

Pathway analysis further refined these insights, revealing MGA-specific metabolic and signaling rewiring. As a core subtype of MGAs, the metabolic abnormalities associated with MCD are more focused on the disruption of fatty acid metabolism, whereas the MGA group exhibits overall inhibition of the metabolic pathway. This reflects a unique regulatory pattern. Specifically, the downregulation of metabolic pathways and the PPAR signaling pathway in the MGA group may be mechanistically homologous to the mechanisms associated with diabetic kidney disease (DKD). In DKD, insufficient activation of the PPAR signaling pathway leads to lipid metabolism disorders and impaired fatty acid oxidation, a process analogous to the pathway downregulation observed in MGAs [32, 33]. Moreover, the overall inhibition of metabolic pathways in the MGA group aligns with the downregulation of the fatty acid β-oxidation pathway in lupus nephritis (LN), and oxidative phosphorylation and tricarboxylic acid cycle pathways in autosomal dominant polycystic kidney disease, suggesting that such pathway regulation may be associated with energy metabolism imbalance in renal cells [22, 34], underscoring a shared pathogenic thread across these kidney diseases. Interestingly, the regulatory direction can be disease specific, as the inhibition of metabolic pathways in MGAs contrasts with the activation of fatty acid metabolism in MN, highlighting the complexity of metabolic regulation [29]. Moreover, the inhibition of protein digestion and absorption pathways in MGAs shares similarities with the nutritional metabolic reprogramming that supports cyst proliferation in autosomal dominant polycystic kidney disease, suggesting that coordinated regulation of substance absorption and metabolism may represent a potential adaptive response to renal injury [35]. In addition to metabolism, the downregulation of the renin–angiotensin system in the MGA group strongly contrasts with its overactivation in DKD, indicating disease-specific differences in renal hemodynamic regulation [32].

Conversely, activated basal transcription factors and nucleotide excision repair pathways form a coordinated regulatory module. Studies have revealed the activated transcription of cell-death-related genes and a significant increase in the activity of DNA damage repair-related pathways in MN [29]. Similarly, in MGAs, both the nucleotide excision repair pathway and the basic transcription factor pathway are upregulated. The former ensures the stability of genetic material, whereas the latter provides crucial support for the initiation of gene transcription. Both collectively contribute to the molecular response to injury, which indicates that the activation of transcription- and DNA repair-related pathways may represent a conserved adaptive response of kidney diseases to injury. The specific remodeling of the aforementioned pathways ultimately converges on 13 core upregulated nuclear proteins with high DRs. As key nodes in these pathways, the functional abnormalities of these proteins may serve as the core drivers of the pathological mechanisms underlying MGAs.

Notably, several of these proteins have established links to renal pathology. YY1 is highly expressed in MGAs, a phenomenon also observed in other renal diseases. In LN, the expression of YY1 is significantly upregulated, promoting the occurrence and development of the disease by regulating the secretion of inflammatory factors. Targeted inhibition of its expression can reduce the release of inflammatory factors and alleviate local inflammatory responses in the kidneys [36, 37]. In addition, in DKD, YY1 participates in the pathological process as a fibrosis regulatory factor. On the one hand, it mediates abnormal glycolysis and renal fibrosis by activating the estimated glomerular filtration rate/PKM2/HIF-1α pathway. On the other hand, its deacetylation modification can inhibit the epithelial–mesenchymal transition of renal tubular cells to delay the progression of the disease [38–40]. Taken together, these findings suggest that the upregulation of YY1 in MGAs may exacerbate renal inflammation through pro-inflammatory mechanisms. In AKI, YY1 exhibits a protective effect. Its down-regulation leads to an increase in KIM1 gene expression, which promotes apoptosis and inflammatory responses in renal tubular cells and aggravates kidney damage. Activating YY1 or specifically knocking out KIM1 in renal tubules can significantly alleviate kidney damage in AKI mouse models [41]. The specific mechanism of YY1 in MGAs still needs verification. TAF9, a key factor for RNA polymerase II-mediated transcription initiation, is closely related to cytomegalovirus-induced LN [42, 43]. It can trigger TAF9-specific immune responses, leading to proteinuria and pathological changes in glomerulonephritis in mice [43]. In MGAs, the upregulation of TAF9 may exacerbate renal tissue inflammatory responses and functional disorders by inducing immune reactions. RFC1 is a key folate transporter that located to the basolateral membranes of renal tubules, and its well-characterized role as a core molecular target for methotrexate highlights its strong translational relevance [44]. Methotrexate is a folate derivative that inhibits several enzymes responsible for nucleotide synthesis. This inhibition leads to suppression of inflammation as well as prevention of cell division. Methotrexate is an antineoplastic agent clinically approved for treating a broad spectrum of cancers, including acute lymphoblastic leukemia, acute promyelocytic leukemia, bladder cancer, breast cancer, and CNS lymphoma, as well as autoimmune diseases such as severe rheumatoid arthritis (https://go.drugbank.com/drugs/DB00563). In renal pathophysiology, RFC1 expression is tightly linked to renal injury: in AKI, reduced RFC1 expression correlates with diminished plasma folate levels, which in turn show a negative association with plasma creatinine concentrations. Extending this understanding, we speculate that the upregulation of RFC1 observed in MGAs represents an adaptive compensatory renal mechanism, serving to enhance folate transport and mitigate kidney injury [45]. This finding provides a rationale for exploring RFC1-directed strategies—such as repurposing methotrexate, to manage MGA-associated renal dysfunction, linking our basic research observations to actionable clinical implications.

As a subunit of RNA polymerases I and III, POLR1D is involved in ribosomal RNA synthesis, and its dysregulation is associated with a variety of diseases [46]. Previous studies have demonstrated that the overexpression of POLR1D in human cells can activate mammalian target of rapamycin complex 1 (mTORC1), thereby promoting renal interstitial inflammation and fibrosis [22, 47, 48]. On this basis, we hypothesize that this mechanism may also play a role in the pathological process of MGAs. WDR3 is a core hub gene for post-renal transplantation AKI, and its abnormal expression is involved in the early injury of transplanted kidneys [49]. In the present study, WDR3 was upregulated in MGAs, suggesting that WDR3 may serve as a potential common biomarker for both MGAs and post-renal transplantation AKI. AK6 is involved in nucleotide metabolism, maintaining intracellular ATP homeostasis while possessing antioxidant functions to scavenge reactive oxygen species [50]. Whether the upregulation of AK6 in MGAs exerts an antioxidant protective effect requires further investigation. MBD3, BCL7C, SMARCD1, and several other upregulated nuclear proteins identified in our study are related to tumor occurrence and development. The upregulation of these proteins in MGAs does not pose a carcinogenic risk, but it provides a new perspective for understanding the regulation of cell homeostasis in MGAs. MBD3 facilitates gastric cancer cell proliferation through the phosphatidylinositol 3-kinase-Akt pathway, a key driver of diabetic nephropathy fibrosis [49]. However, its upregulation in MGAs fails to trigger fibrosis, likely owing to the absence of a high-glucose microenvironment, and may only sustain basic cell proliferation in the MGA microenvironment. BCL7C and SMARCD1, both members of the SWI/SNF that exerts functions via chromatin structure regulation, are upregulated in MGAs and exhibit completely opposite functional properties. BCL7C exerts a tumor-suppressive effect by negatively regulating the Wnt and apoptotic pathways to inhibit carcinogenesis, and its upregulation may engage in MGAs injury response through the suppression of excessive mesangial cell proliferation [51, 52]. SMARCD1 promotes tumor proliferation by regulating target gene transcription, and its upregulation is highly likely to facilitate the pathological progression of MGAs via chromatin remodeling [53, 54]. These opposing regulatory effects imply that the pathological process of MGAs may stem from the balance of multiple chromatin remodeling factors with opposite functions, and the specific mechanisms involved require further in-depth research. The highly conserved RPP30 is regulated by tRNA to promote tumor development and angiogenesis [55, 56]. Whether it promotes angiogenesis and participates in inflammation in MGAs needs to be verified, specifically on the basis of the pathological characteristics of MGAs. Similarly, ACTR6 is highly expressed in hepatocellular carcinoma and is linked to poor prognosis. It may promote hepatocarcinogenesis by regulating the cell cycle to induce immune cell infiltration, and its potential role in regulating cell proliferation and inflammation in MGAs needs targeted exploration [57]. GTF2A2, a key factor for RNA polymerase II-mediated transcription initiation, is upregulated in osteosarcoma and multiple myeloma and causes inflammatory molecule dysregulation [58, 59]. Its upregulation in MGAs may exacerbate renal inflammation via activating inflammatory pathways, although this requires further verification. As a member of the TIP60–p400 complex, DMAP1 inhibits transcription via DNA methylation and contributes to DNA damage repair and tumor suppression, but the specific association between its upregulation and the pathological processes of MGAs has yet to be investigated [60, 61].

**Figure 6:**
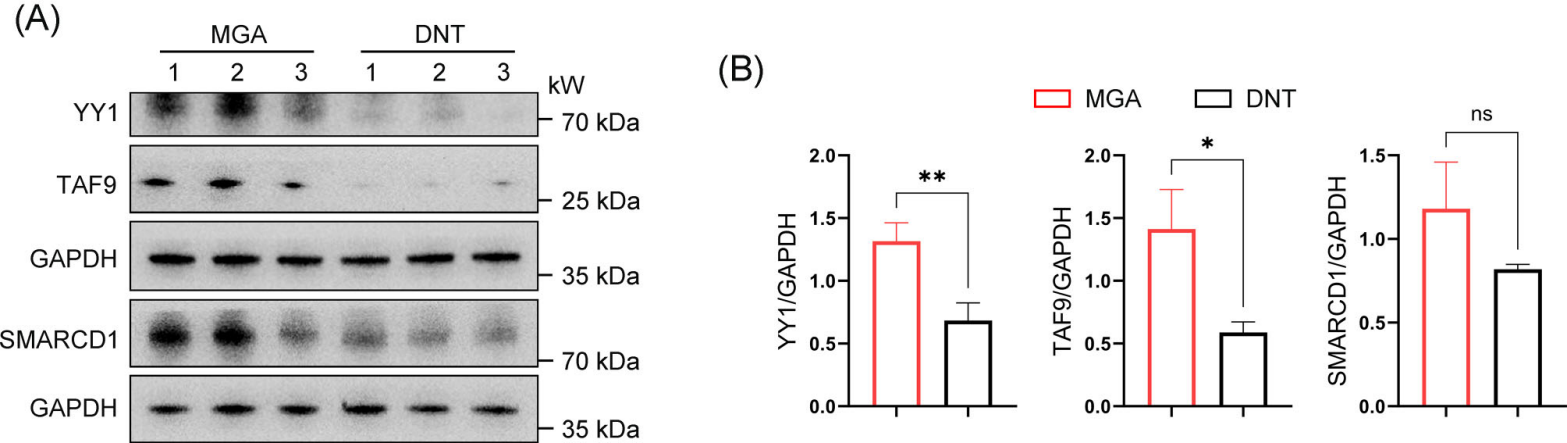
Western blot analysis and quantification of YY1, TAF9, and SMARCD1 expression in renal tissues (GAPDH served as loading control) (*n* = 3). Data are presented as mean ± SD. * *P* < 0.05, **P < 0.01, ns *P* > 0.05.

Collectively, the prioritized candidates from our proteomic screen, such as YY1 and POLR1D, are not merely differentially expressed but embody key “druggable” nodes within the dysregulated molecular network of MGAs. The upregulation of YY1, a pleiotropic transcription factor implicated in renal inflammation and fibrosis in LN and DKD, positions it as a master regulator potentially driving similar pathogenic processes in MGAs. Its established “druggable” profile opens avenues for exploring repurposed or novel YY1 inhibitors in experimental models of MGA. Similarly, the association of POLR1D with mTORC1 activation provides a direct link to a therapeutically tractable pathway. While mTOR inhibitors like sirolimus have known renal applications (e.g. in transplantation and some glomerulopathies), our data suggested a rationale for evaluating their efficacy specifically in the subset of MGAs characterized by POLR1D upregulation [62–64]. Beyond these examples, other upregulated proteins like TAF9 (linked to immune activation) and RFC1 (a known drug target for methotrexate) further enrich the pool of mechanistically grounded, actionable targets (Fig. 5) [65]. This target-centric framework moves beyond descriptive biomarker identification and provides a hypothesis-driven roadmap for subsequent functional validation and therapeutic exploration. In independent validation, this significant upregulation of core candidates like YY1 and TAF9 has been confirmed at the protein level in an independent cohort (Fig. 6), strengthening confidence in their role. Therefore, we propose that YY1 and POLR1D represent prioritized, mechanistically grounded targets for MGAs. Future work should focus on *in vivo* validation using genetic or pharmacological inhibition in relevant models to confirm their causal role and therapeutic efficacy.

From a clinical application perspective, the diagnosis of MGAs still relies on pathological morphological examination, with a lack of specific markers to assist in pathological diagnosis. In contrast, other renal diseases have established clear marker systems. For example, LN employs anti-double-stranded DNA antibodies and complement C3/C4 levels as monitoring indicators [57]. DKD can be monitored using urinary cystatin C to predict its occurrence and progression [66], while AKI uses neutrophil gelatinase-associated lipocalin as a biomarker [66]. It is also notable that MCD possesses well-defined markers including urine serpin family A member 1 (SERPINA1), CD80, and CD14, along with plasma SERPIN family protein profiles, that help distinguish it from other renal diseases, and urine CD80 can reflect disease activity [29, 67]. However, as a broad category encompassing multiple lesions and other conditions, MGAs still exhibit significant gaps in tissue marker research. This prevents renal biopsy samples from exerting molecular diagnostic value, thereby limiting research on the pathological mechanisms of MGAs and the development of precise diagnostic methods. Therefore, the screening of 13 significantly upregulated nuclear proteins in renal tissue in this study is necessary. On the one hand, it can fill the gap in the search for MGA tissue markers; on the other hand, these tissue nuclear proteins can also provide potential targets for MGA intervention. By clarifying their associations with the pathological mechanisms of MGA tissue, this study lays a foundation for subsequent mechanistic verification and clinical translation. While the current research provides a deep proteomic mapping of renal tissue in MGAs, a critical future direction involves translating these findings into clinically accessible biomarkers. Future studies should systematically integrate multi-sample profiling, particularly non-invasive liquid biopsies like urine and serum proteomics. Such efforts will be essential to determine whether the tissue-derived signatures (e.g. the 13 upregulated nuclear proteins) have detectable correlation in bodily fluids, and to discover novel urine-specific diagnostic or prognostic markers for MGAs. Establishing a correlation between tissue pathology and urinary proteomic profiles represents a vital step towards developing non-invasive tools for diagnosis, risk stratification, and treatment monitoring in patients with MGAs.

## Ethics statement

This study was conducted in strict accordance with the ethical principles of the Declaration of Helsinki and relevant national and institutional regulations for medical research involving human subjects. All procedures related to the collection, handling, and analysis of human kidney tissue samples were reviewed and approved by the Ethics Committee of West China Hospital, Sichuan University [Approval No. 2024(1221)].

## Supplementary Material

pbag006_Supplemental_Files

## Data Availability

The mass spectrometry data have been deposited to the Proteome Xchange Consortium via the iProX partner repository with the dataset identifier PXD070517.

## References

[1] Perkowska-Ptasinska A, Bartczak A, Wagrowska-Danilewicz M et al. Clinicopathologic correlations of renal pathology in the adult population of Poland. Nephrol Dial Transplant. 2017;32:ii209–18. 10.1093/ndt/gfw365 28339709

[2] Kim BS, Kim YK, Shin YS et al. Natural history and renal pathology in patients with isolated microscopic hematuria. Korean J Intern Med. 2009;24:356–61. 10.3904/kjim.2009.24.4.356 19949735 PMC2784980

[3] López-Gómez JM, Rivera F. Spanish registry of glomerulonephritis 2020 revisited: past, current data and new challenges. Nefrología. 2020;40:371–83. 10.1016/j.nefro.2020.04.012 32646677

[4] Slugen I, Danis D, Nyitrayová O et al. Minor glomerular abnormalities in chronic tubulointerstitial nephritis. Vnitr Lek. 1994;40:757–9. 7839626

[5] Yu BC, Cho NJ, Park S et al. Minor glomerular abnormalities are associated with deterioration of long-term kidney function and mitochondrial injury. JCM. 2019;9:33. 10.3390/jcm9010033 31877839 PMC7019622

[6] Cai F, Gu Y, Ling Y et al. Proteomics in pancreatic cancer. Biomark Res. 2025;13:93. 10.1186/s40364-025-00805-y 40619414 PMC12232871

[7] Yi G, Luo H, Zheng Y et al. Exosomal proteomics: unveiling novel insights into lung cancer. Aging Dis. 2024;16:876–900. 10.14336/ad.2024.0409 38607736 PMC11964432

[8] Zhao Y, Xue Q, Wang M et al. Evolution of mass spectrometry instruments and techniques for blood proteomics. J Proteome Res. 2023;22:1009–23. 10.1021/acs.jproteome.3c00102 36932955

[9] Yang Y, Zhang Y, Li Y et al. Complement classical and alternative pathway activation contributes to diabetic kidney disease progression: a glomerular proteomics on kidney biopsies. Sci Rep. 2025;15:495. 10.1038/s41598-024-84900-4 39753879 PMC11698715

[10] Guo T, Kouvonen P, Koh CC et al. Rapid mass spectrometric conversion of tissue biopsy samples into permanent quantitative digital proteome maps. Nat Med. 2015;21:407–13. 10.1038/nm.3807 25730263 PMC4390165

[11] Livingston MJ, Shu S, Fan Y et al. Tubular cells produce FGF2 via autophagy after acute kidney injury leading to fibroblast activation and renal fibrosis. Autophagy. 2023;19:256–77. 10.1080/15548627.2022.2072054 35491858 PMC9809951

[12] Fu J, Sun Z, Wang X et al. The single-cell landscape of kidney immune cells reveals transcriptional heterogeneity in early diabetic kidney disease. Kidney Int. 2022;102:1291–304. 10.1016/j.kint.2022.08.026 36108806 PMC9691617

[13] Xu S, Yang X, Chen Q et al. Leukemia inhibitory factor is a therapeutic target for renal interstitial fibrosis. EBioMedicine. 2022;86:104312. 10.1016/j.ebiom.2022.104312 36335669 PMC9646860

[14] Jiang W, Liu M, Su T et al. GlycoPCT: pressure cycling technology-based quantitative glycoproteomics reveals distinctive N-glycosylation in human liver biopsy samples of nonalcoholic fatty liver disease. J Proteome Res. 2025;24:202–9. 10.1021/acs.jproteome.4c00588 39600157

[15] Cai X, Xue Z, Wu C et al. High-throughput proteomic sample preparation using pressure cycling technology. Nat Protoc. 2022;17:2307–25. 10.1038/s41596-022-00727-1 35931778 PMC9362583

[16] Shen SS, Ro JY. Histologic diagnosis of renal mass biopsy. Arch Pathol Lab Med. 2019;143:705–10. 10.5858/arpa.2018-0272-RA 30969154

[17] Nakach M, Authelin JR, Perrin MA et al. Comparison of high pressure homogenization and stirred bead milling for the production of nano-crystalline suspensions. Int J Pharm. 2018;547:61–71. 10.1016/j.ijpharm.2018.05.042 29787896

[18] Nitsos C, Filali R, Taidi B et al. Current and novel approaches to downstream processing of microalgae: A review. Biotechnol Adv. 2020;45:107650. 10.1016/j.biotechadv.2020.107650 33091484

[19] Bao K, Li X, Kajikawa T et al. Pressure cycling technology assisted mass spectrometric quantification of gingival tissue reveals proteome dynamics during the initiation and progression of inflammatory periodontal disease. Proteomics. 2020;20:e1900253. 10.1002/pmic.201900253 31881116 PMC7033018

[20] Kondo A, McGrady M, Nallapothula D et al. Spatial proteomics of human diabetic kidney disease, from health to class III. Diabetologia. 2024;67:1962–79. 10.1007/s00125-024-06210-8 39037603

[21] Bărar AA, Pralea IE, Maslyennikov Y et al. Minimal change disease: pathogenetic insights from glomerular proteomics. Int J Mol Sci. 2024;25:5613. 10.3390/ijms25115613 38891801 PMC11171934

[22] Mao Z, Tan Y, Tao J et al. Renal mTORC1 activation is associated with disease activity and prognosis in lupus nephritis. Rheumatology (Oxford). 2022;61:3830–40. 10.1093/rheumatology/keac037 35040950 PMC9608003

[23] Si S, Liu H, Xu L et al. Identification of novel therapeutic targets for chronic kidney disease and kidney function by integrating multi-omics proteome with transcriptome. Genome Med. 2024;16:84. 10.1186/s13073-024-01356-x 38898508 PMC11186236

[24] Zhao Z, Hu Z, Zeng R et al. HMGB1 in kidney diseases. Life Sci. 2020;259:118203. 10.1016/j.lfs.2020.118203 32781069

[25] Levstek T, Trebušak Podkrajšek K. Telomere attrition in chronic kidney diseases. Antioxidants. 2023;12:579. 10.3390/antiox12030579 36978826 PMC10045531

[26] Schwab SK, Harris PS, Michel C et al. Quantifying protein acetylation in diabetic nephropathy from Formalin-fixed paraffin-embedded tissue. Proteomics Clinical Apps. 2024;18:e202400018. 10.1002/prca.202400018 PMC12291557 38923810

[27] Norén Å, Boi R, Pullerits R et al. Proteomic analysis of human kidney biopsies unveils emerging acute kidney injury very early after liver graft reperfusion. J Transl Med. 2025;23:658. 10.1186/s12967-025-06695-w 40524147 PMC12172208

[28] Huang Q, Fei X, Zhong Z et al. Stratification of diabetic kidney diseases via data-independent acquisition proteomics-based analysis of human kidney tissue specimens. Front Endocrinol. 2022;13:995362. 10.3389/fendo.2022.995362 PMC9714485 36465646

[29] Muruve DA, Debiec H, Dillon ST et al. Serum protein signatures using aptamer-based proteomics for minimal change disease and membranous nephropathy. Kidney International Reports. 2022;7:1539–56. 10.1016/j.ekir.2022.04.006 35812291 PMC9263421

[30] Wang D, Wu C, Chen S et al. Urinary complement profile in IgA nephropathy and its correlation with the clinical and pathological characteristics. Front Immunol. 2023;14:1117995. 10.3389/fimmu.2023.1117995 37020564 PMC10068869

[31] Hou YP, Diao TT, Xu ZH et al. Bioinformatic analysis combined with experimental validation reveals novel hub genes and pathways associated with focal segmental glomerulosclerosis. Front Mol Biosci. 2021;8:691966. 10.3389/fmolb.2021.691966 35059432 PMC8763695

[32] Wei L, Gao J, Wang L et al. Multi-omics analysis reveals the potential pathogenesis and therapeutic targets of diabetic kidney disease. Hum Mol Genet. 2024;33:122–37. 10.1093/hmg/ddad166 37774345

[33] Zhao J, He K, Du H et al. Bioinformatics prediction and experimental verification of key biomarkers for diabetic kidney disease based on transcriptome sequencing in mice. PeerJ. 2022;10:e13932. 10.7717/peerj.13932 36157062 PMC9504448

[34] Song X, Pickel L, Sung HK et al. Reprogramming of energy metabolism in human PKD1 polycystic kidney disease: a systems biology analysis. Int J Mol Sci. 2024;25:7173. 10.3390/ijms25137173 39000280 PMC11240917

[35] Cornec-Le Gall E, Alam A, Perrone RD. Autosomal dominant polycystic kidney disease. The Lancet. 2019;393:919–35. 10.1016/s0140-6736(18)32782-x 30819518

[36] Yuan H, Peng Z, Li H et al. Oxymatrine ameliorates lupus nephritis by targeting the YY1-mediated IL-6/STAT3 axis. Int J Mol Sci. 2024;25:12260. 10.3390/ijms252212260 39596325 PMC11594375

[37] Wang B, Jiang X, Li Y et al. YY1 alleviates lupus nephritis-induced renal injury by reducing the Th17/Treg cell ratio via the IFN-γ/Fra2 axis. Lab Invest. 2022;102:872–84. 10.1038/s41374-022-00777-9 35361881

[38] Li X, Ma TK, Wang M et al. YY1-induced upregulation of LncRNA-ARAP1-AS2 and ARAP1 promotes diabetic kidney fibrosis via aberrant glycolysis associated with EGFR/PKM2/HIF-1α pathway. Front Pharmacol. 2023;14:1069348. 10.3389/fphar.2023.1069348 36874012 PMC9974832

[39] Yang TT, Shao YT, Cheng Q et al. YY1/HIF-1α/mROS positive-feedback loop exacerbates glomerular mesangial cell proliferation in mouse early diabetic kidney disease. Acta Pharmacol Sin. 2025;46:1974–89. 10.1038/s41401-025-01498-7 40038466 PMC12205067

[40] Du L, Qian X, Li Y et al. Sirt1 inhibits renal tubular cell epithelial-mesenchymal transition through YY1 deacetylation in diabetic nephropathy. Acta Pharmacol Sin. 2021;42:242–51. 10.1038/s41401-020-0450-2 32555442 PMC8027604

[41] Yang C, Xu H, Yang D et al. A renal YY1-KIM1-DR5 axis regulates the progression of acute kidney injury. Nat Commun. 2023;14:4261. 10.1038/s41467-023-40036-z 37460623 PMC10352345

[42] Jin C, Dong B, Xie Y et al. LncRNA938/TAF9/TTK axis promotes EMT and serves as a therapeutic target in hepatoblastoma. J Transl Med. 2025;23:946. 10.1186/s12967-025-06809-4 40841910 PMC12372275

[43] Hsieh AH, Kuo CF, Chou IJ et al. Human cytomegalovirus pp65 peptide-induced autoantibodies cross-reacts with TAF9 protein and induces lupus-like autoimmunity in BALB/c mice. Sci Rep. 2020;10:9662. 10.1038/s41598-020-66804-1 32541894 PMC7295797

[44] Inoue K, Yuasa H. Molecular basis for pharmacokinetics and pharmacodynamics of methotrexate in rheumatoid arthritis therapy. Drug Metab Pharmacokinet. 2014;29:12–9. 10.2133/dmpk.dmpk-13-rv-119 24284432

[45] Wang Y, Zhao R, Russell RG et al. Localization of the murine reduced folate carrier as assessed by immunohistochemical analysis. Biochimica et Biophysica Acta (BBA)—Biomembranes. 2001;1513:49–54. 10.1016/s0005-2736(01)00340-6 11427193

[46] Palumbo RJ, Belkevich AE, Pascual HG et al. A clinically-relevant residue of POLR1D is required for Drosophila development. Dev Dyn. 2022;251:1780–97. 10.1002/dvdy.505 35656583 PMC10723622

[47] Gorjão N, Borowski LS, Szczesny RJ et al. POLR1D, a shared subunit of RNA polymerase I and III, modulates mTORC1 activity. Biochimica et Biophysica Acta (BBA)—Molecular Cell Research. 2025;1872:119957. 10.1016/j.bbamcr.2025.119957 40222657

[48] Ling Y, Cai F, Su T et al. Glycosylation in kidney diseases. Precision clinical medicine. 2025;8:pbaf017. 10.1093/pcmedi/pbaf017 40852041 PMC12368498

[49] Bi H, Zhang M, Wang J et al. The mRNA landscape profiling reveals potential biomarkers associated with acute kidney injury AKI after kidney transplantation. PeerJ. 2020;8:e10441. 10.7717/peerj.10441 33312771 PMC7703406

[50] Sodders MJ, Avila-Pacheco J, Okorie EC et al. Genetic screening and metabolomics identify glial adenosine metabolism as a therapeutic target in Parkinson’s disease. BioRxiv. 2024. 10.1101/2024.05.15.594309

[51] Huang C, Hao Q, Shi G et al. BCL7C suppresses ovarian cancer growth by inactivating mutant p53. J Mol Cell Biol. 2021;13:141–50. 10.1093/jmcb/mjaa065 33306126 PMC8104935

[52] Uehara T, Kage-Nakadai E, Yoshina S et al. The tumor suppressor BCL7B functions in the Wnt signaling pathway. PLoS Genet. 2015;11:e1004921. 10.1371/journal.pgen.1004921 25569233 PMC4287490

[53] Fu G, Liu Y, Qian C et al. SMARCD1 is a dual regulator of PD-L1 expression and cell proliferation facilitating tumor evasion. Pathology—Research and Practice. 2025;270:155975. 10.1016/j.prp.2025.155975 40228401

[54] Ross C, Gong LY, Jenkins LM et al. SMARCD1 is a “Goldilocks” metastasis modifier. BioRxiv. 2024. 10.1101/2024.01.24.577061

[55] Li G, Zhai Y, Liu H et al. RPP30, a transcriptional regulator, is a potential pathogenic factor in glioblastoma. Aging. 2020;12:16155–71. 10.18632/aging.103596 32702667 PMC7485703

[56] Kan Y, Lu X, Feng L et al. RPP30 is a novel diagnostic and prognostic biomarker for gastric cancer. Front Genet. 2022;13:888051. 10.3389/fgene.2022.888051 35928448 PMC9343801

[57] Wang J, Song M, Tang J et al. Expression, prognosis and preliminary investigation of the mechanism of action of ACTR6, a member of the ARPs gene family, in hepatocellular carcinoma. Front Med. 2025;12:1513233. 10.3389/fmed.2025.1513233 PMC11931126 40130245

[58] Wang G, Zhang Z, Yang M et al. Comparative proteomics analysis of human osteosarcoma by 2D DIGE with MALDI-TOF/TOF MS. Journal of Bone Oncology. 2016;5:147–52. 10.1016/j.jbo.2016.05.002 28008374 PMC5154703

[59] Wan Y, Jiang J, Chen M et al. Unravelling the imbalanced Th17-like cell differentiation by single-cell RNA sequencing in multiple myeloma. Int Immunopharmacol. 2023;124:110852. 10.1016/j.intimp.2023.110852 37657245

[60] Negishi M, Chiba T, Saraya A et al. Dmap1 plays an essential role in the maintenance of genome integrity through the DNA repair process. Genes Cells. 2009;14:1347–57. 10.1111/j.1365-2443.2009.01352.x 19845771

[61] Rojas S, Barghouth PG, Karabinis P et al. The DNA methyltransferase DMAP1 is required for tissue maintenance and planarian regeneration. Dev Biol. 2024;516:196–206. 10.1016/j.ydbio.2024.08.007 39179016 PMC11521571

[62] Ma MKM, Yung S, Chan TM. mTOR inhibition and kidney diseases. Transplantation. 2018;102:S32–40. 10.1097/tp.0000000000001729 29369972

[63] Rangan GK, Nguyen T, Mainra R et al. Therapeutic role of sirolimus in non-transplant kidney disease. Pharmacol Ther. 2009;123:187–206. 10.1016/j.pharmthera.2009.03.014 19374918

[64] Wang F, Yang C, Shu Y et al. Safety and efficacy of sirolimus combined with cyclosporine in primary membranous nephropathy: a randomized controlled trial. BMC Med. 2025;23:323. 10.1186/s12916-025-04173-0 40442735 PMC12123714

[65] Kung TN, Dennis J, Ma Y et al. RFC1 80G>A is a genetic determinant of methotrexate efficacy in rheumatoid arthritis: a human genome epidemiologic review and meta-analysis of observational studies. Arthritis Rheumatol. 2014;66:1111–20. 10.1002/art.38331 24782176

[66] Wang N, Lu Z, Zhang W et al. Serum cystatin C trajectory is a marker associated with diabetic kidney disease. Front Endocrinol. 2022;13:824279. 10.3389/fendo.2022.824279 PMC9130469 35634510

[67] Mishra OP . Urinary CD 80 in nephrotic syndrome: a biomarker to distinguish minimal change disease from other glomerular diseases. Kidney International Reports. 2020;5:1851–2. 10.1016/j.ekir.2020.09.027 33165400 PMC7610000

