# Supplementary material for: Pressure cycling technology-assisteddata-independent acquisition proteomics reveals molecular alterations and potential therapeutic targets in minor glomerular abnormalities
Source: Precis Clin Med. 2026 Feb 13;9(1):pbag006. doi: 10.1093/pcmedi/pbag006 (PMC12999293; doi:10.1093/pcmedi/pbag006)
Supplement: pbag006_Supplemental_Files [file pbag006_supplemental_files.zip › Supplementary data.docx]

**Supplementary data**

**Supplementary Tables**

**Supplementary Table 1.** Baseline characteristics of patients with MGA and DNT.

**Supplementary Table 2.** Quantified PGs of HeLa cell lysate (QC sample) across six replicates.

**Supplementary Table 3.** Quantified PGs using PCT-assisted DIA proteomics.

**Supplementary Table 4.** Quantified DEPGs using PCT-assisted DIA proteomics.

**Supplementary Figure 1.** Histopathological evaluation of kidney tissues from the study cohorts.

**Supplementary Figure 2.** Base peak intensity (BPI) chromatograms of the six QC sample datasets (A) and Pearson correlation heatmap of quantified protein expression levels across six replicates (B).

**Supplementary Figure 3.** Pearson correlation heatmap of quantified protein expression levels across all samples.

**Supplementary Figure 4.** Pearson correlation heatmap of quantified 13 upregulated nuclear proteins and clinical parameters.

**
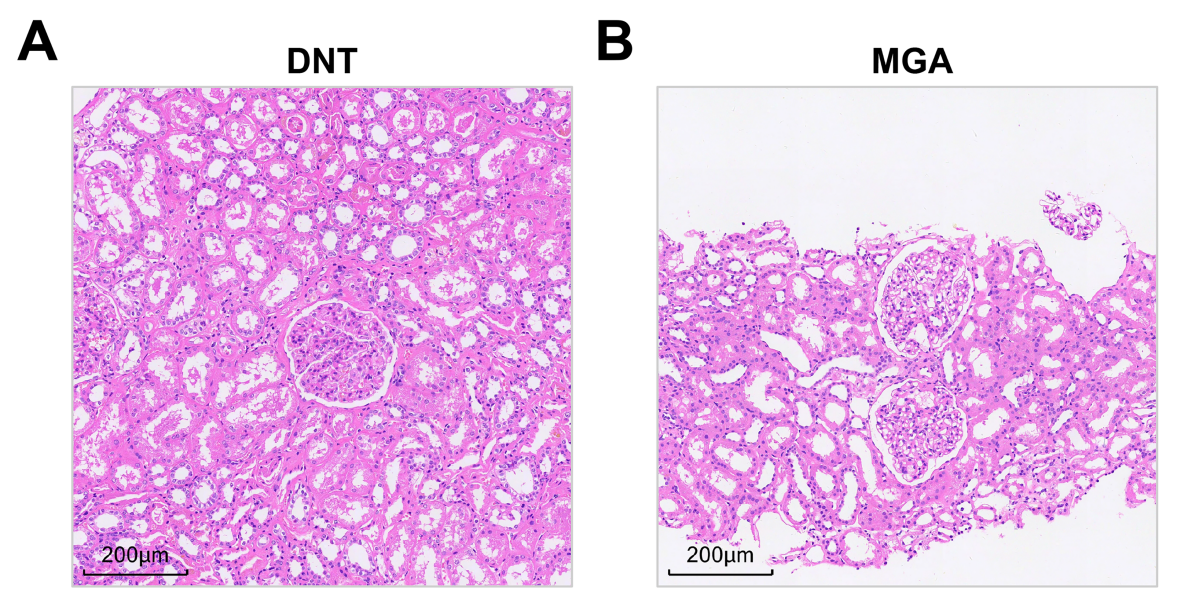
**

**Figure 1.** Histopathological evaluation of kidney tissues from the study cohorts. (**A**) H&E staining of DNT renal tissue from a patient undergoing nephrectomy for a renal space-occupying lesion. The renal parenchyma shows preserved architecture in both glomeruli and tubules, with no evidence of tumor cell infiltration (representative image, 50x magnification). (**B**) H&E staining of renal tissue from a patient with MGA. Glomerular architecture is largely preserved, without significant mesangial hypercellularity, capillary wall thickening, or glomerulosclerosis (representative image, 50x magnification). Scale bar: 200 μm.


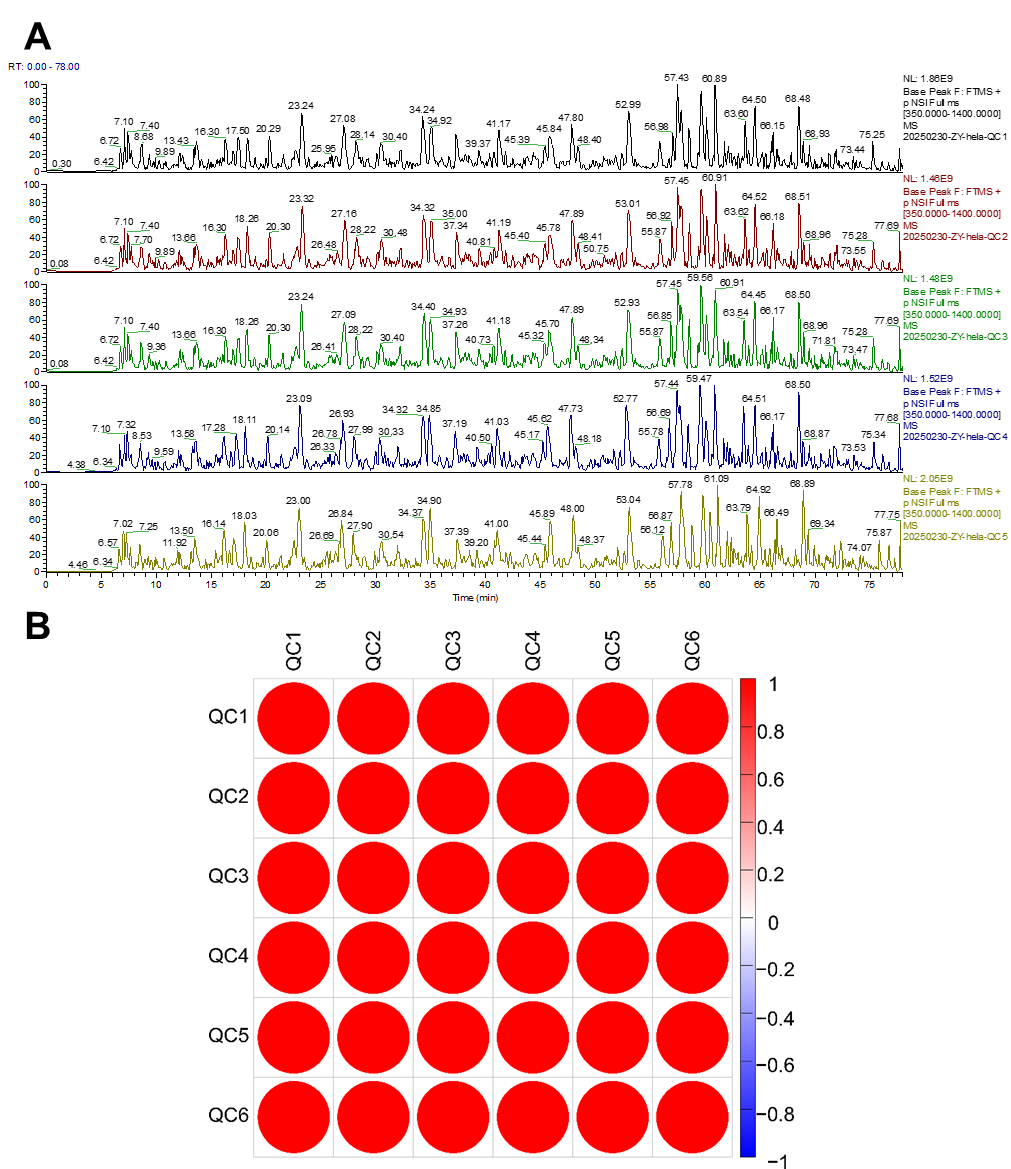


**Supplementary Figure 2.** Base peak intensity (BPI) chromatograms of the six QC sample datasets (**A**) and Pearson correlation heatmap of quantified protein expression levels across six replicates (**B**).


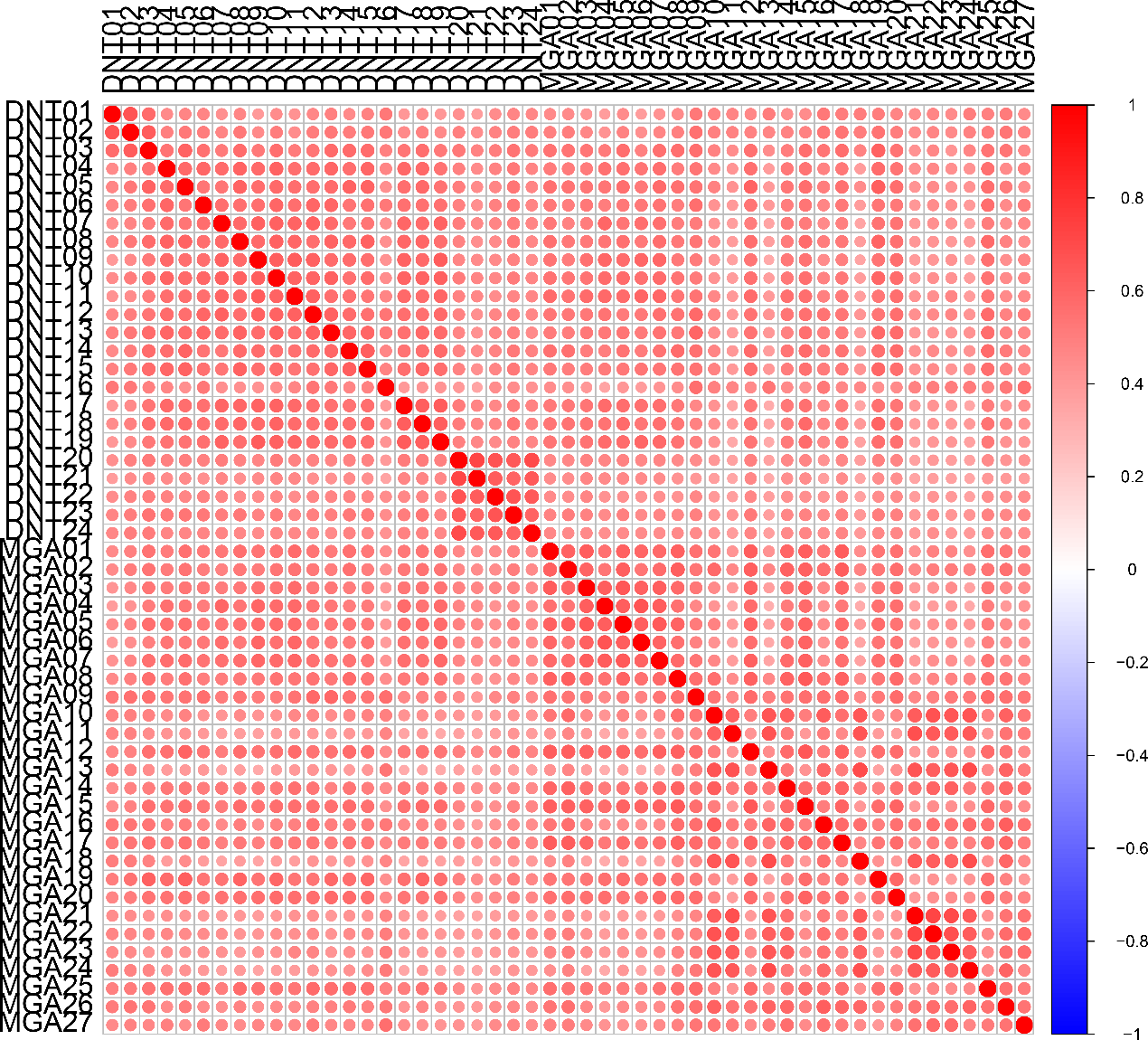


**Supplementary Figure 3.** Pearson correlation heatmap of quantified protein expression levels across all samples.


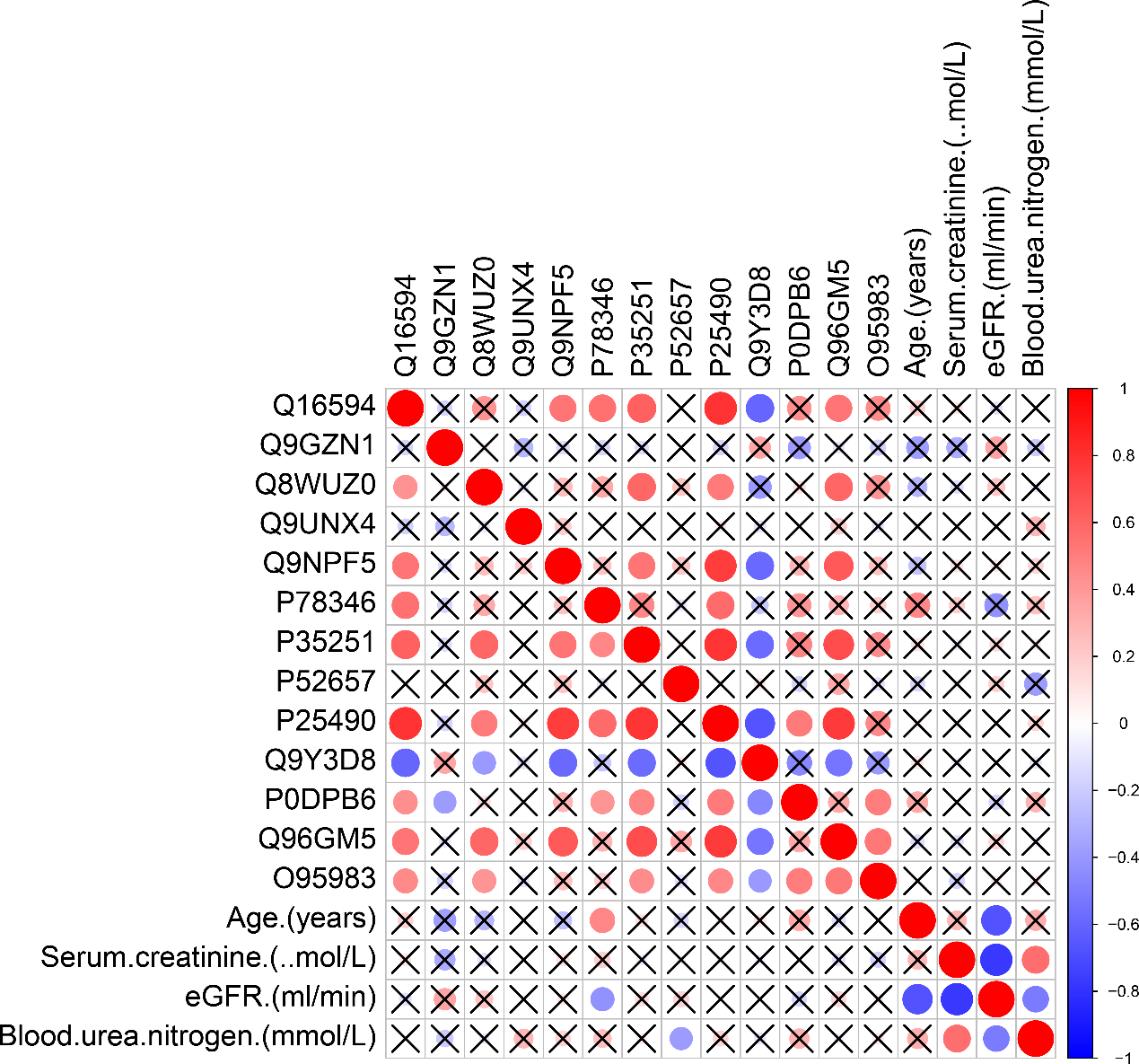


**Supplementary Figure 4.** Pearson correlation heatmap of quantified 13 upregulated nuclear proteins and clinical parameters.
